# Editorial: Perceptual Motor Control in the Virtual Environment and Its Implications in the Real World

**DOI:** 10.3389/fspor.2023.1138516

**Published:** 2023-02-06

**Authors:** Hirofumi Ida, Kazunobu Fukuhara, Sambit Mohapatra, Hirofumi Sekiguchi

**Affiliations:** ^1^ Department of Sports and Health Management, Jobu University, Isesaki, Japan; ^2^ Department of Health Promotion Science, Tokyo Metropolitan University, Hachioji, Japan; ^3^ Department of Rehabilitation and Movement Science, The University of Vermont, Burlington, VT, United States; ^4^ Graduate Faculty of Interdisciplinary Research, University of Yamanashi, Kofu, Japan

**Keywords:** virtual reality, physical reality, visuomotor control, perception-action coupling, sense of presence, perceptual distortion

**Editorial on the Research Topic** Perceptual Motor Control in the Virtual Environment and Its Implications in the Real World.

Virtual reality (VR) has become widely utilized for studying human perceptual-motor control. In fact, a search of PubMed using the terms “virtual reality” and “rehabilitation” uncovered an increasing number of relevant publications from 248 in 2017 to 564 in 2022, a 2.27 times increase. Using the terms “virtual reality” and “sports” revealed an increase from 25 to 111 over the same 5-year period, a 4.44 times increase. This implies that VR display techniques have been accepted as a valid research tool for neuroscientific studies related to body movement. However, how and to what extent the perceptual-motor response in VR reflects the natural action, activities, and behaviors in the real world, or physical reality (PR), is not well understood (1, 2).

Nowadays, users and researchers benefit from a variety of immersive and interactive VR devices, such as a head-mounted display (HMD), a room-sized multi-wall system called CAVE (3), sensory video games, and many peripheral equipment options. However, in VR, users will experience some visuomotor mismatches: the body is exposed to a physically natural (gravity and air resistance) but optically artificial (depth information and field of view) environment, and to make matters more complicated, the unnatural virtual scene may modulate the psychological state (sense of presence, perceptual distortion, and visual illusion). Moreover, the graphical/pictorial quality of visual input (simple texture, polygon rendering, and photo-real) also possibly affects the perceptual-motor response of the users. This is all still open to discussion.

In this Research Topic, the aim was to provide neuroscientific insights into the questions of how the central nervous system controls body movement in the highly realistic but unnatural environment of VR, what the implications for neuromotor function in PR are, how the sensorimotor performance in VR changes, and whether VR is comparable to PR. To this end, this Research Topic was expected to update recent findings about perceptual-motor control in any kind of VR setting and to practically discuss the application of this in PR. Although there may be positive and negative effects induced by VR, it is important to deal with both of these aspects from a neutral standpoint.

A custom-made VR system that displays a stereoscopic view on a large flat screen was used for the study on walking by Suda et al. The participants were asked to try to walk through an aperture without collision for the pre- and post-training tests in a PR setting and for the intermediate training session in a VR setting. Both younger and older adult participants in the intervention group with enriched feedback showed that the spatial margins in the aperture became smaller after the VR training, while the success rate remained unchanged. On the other hand, in the control group that undertook simple walking training without feedback, neither the spatial margin nor the success rate was improved. These findings suggest that highly demanding training with enriched feedback in VR helps to improve the ability of the users to walk in PR.

Nasu et al. adopted a practical task in a sports situation. They examined softball bat swing when the batter tried to hit the ball thrown by a real pitcher on an outdoor field and by a virtual pitcher presented in an HMD-based original VR system. The temporal discrimination ability was evaluated by the delta onset, which was defined as the difference in the swing onset time between the slow and fast ball pitch conditions. The results showed that there was little difference in the delta onset between these two visual environments, suggesting that the discrimination ability of softball batters in the VR system reflected that in the PR field. However, it was also shown that the VR system induced earlier swing onset with larger variability. This suggests that, at present, there are a few technical problems with the application of VR in demanding activities with high temporal pressure in sports.

The pitcher-batter paradigm was also used by Nakamoto et al. in which verbal reports in an HMD-based VR setting were assessed instead of interactive movement response. Skilled baseball batters viewed an avatar's pitching motion run at different movement speeds (0.7–1.3 times the control motion) and subsequent ball flight (constant speed). Thereafter they evaluated the perceived ball speed relative to the control motion. The results clearly indicated that the perceived ball speed was modulated by the movement speed of the avatar pitcher, and further, this effect was more apparent in the fast ball speed condition than in the slow ball speed condition. In addition, exploratory analyses further suggested that batters with higher skill levels can more effectively integrate the kinematic information of the pitcher and the movement information of ball flight when making judgments about ball speed.

Large-size VR equipment is also available for perceptual motor experiments. Ida et al. utilized a CAVE (3), which typically has room-sized multiple-screen walls and enables whole-body immersion. They examined another sports-related task, namely “catching”, to identify the differences in the environmental effect of VR compared with the equivalent PR on the muscle activity and joint motion of the catching arm. The results showed that shoulder flexion velocity was lower in VR than in PR. Furthermore, electromyography onsets appeared later (closer to the initiation of arm raising) in the two-dimensional VR presentation than in the PR and in the three-dimensional VR. The findings explicitly suggest that the simulation of VR may induce a modulation in the motor responses of interceptive action.

These studies attempted to explore human actions in VR in contrast to PR in the framework of neuroscientific research on perceptual-motor control. Although all of them reported vision-based responses in VR at this time, further studies focusing on other sensorimotor responses are needed to determine how the central nervous system integrates perceptual input and controls motor output. Such challenges will provide deeper insight into how to effectively apply fast-evolving VR technology to human welfare.
